# Antiproliferative Effect of 4-Methylumbelliferone in Epithelial Ovarian Cancer Cells Is Mediated by Disruption of Intracellular Homeostasis and Regulation of PI3K/AKT and MAPK Signaling

**DOI:** 10.3390/pharmaceutics12070640

**Published:** 2020-07-07

**Authors:** Garam An, Sunwoo Park, Minkyoung Lee, Whasun Lim, Gwonhwa Song

**Affiliations:** 1 Institute of Animal Molecular Biotechnology and Department of Biotechnology, College of Life Sciences and Biotechnology, Korea University, Seoul 02841, Korea; damanda22@korea.ac.kr (G.A.); sunwoojump@korea.ac.kr (S.P.); 2 Department of Food and Nutrition, Kookmin University, Seoul 02707, Korea; m2019546@kookmin.ac.kr

**Keywords:** 4-methylumbelliferone, ovarian cancer, proliferation, endoplasmic reticulum stress, calcium homeostasis

## Abstract

Ovarian cancer has a high mortality rate and high resistance to chemotherapy. Thus, many studies are currently assessing the ability of natural products to induce ovarian cancer cell death. A coumarin derivative, 4-methylumbelliferone (4-MU), has been reported to have anti-cancer effects on various cancers, but its effects on ovarian cancer are not fully understood. In this study, we identified the intracellular mechanism underlying the effects of 4-MU on epithelial ovarian cancer cells. Decreased ovarian cancer cell proliferation and an accumulation of cells in the G2/M phase were observed following 4-MU treatment. Moreover, 4-MU interfered with calcium homeostasis; induced endoplasmic reticulum stress in both cell lines; inhibited AKT and S6 phosphorylation; and increased ERK1/2, P38, and JNK phosphorylation. Furthermore, 4-MU and pharmacological inhibitors showed synergic effects in suppressing cell proliferation. Collectively, our current data indicate that antitumor effects of 4-MU could be appropriate for use as a therapeutic agent against epithelial ovarian cancer cells.

## 1. Introduction

Ovarian cancer has lower five-year survival rates (47.6%) than other gynecological cancers, such as breast cancer (89.9%) and uterine cancer (81.2%) [1]. Among the subtypes of ovarian cancer, epithelial ovarian cancer, categorized as either the serous, endometrioid, mucinous, or clear-cell types, accounts for approximately 90% of the cases. There are several reasons that contribute to the low survival rates of ovarian cancer patients. Firstly, most epithelial ovarian cancer cases are diagnosed at above stage III, because they have few symptoms in the early stages [2]. According to the FIGO staging system, when ovarian cancer is at stage III or IV, the carcinoma has metastasized to the retroperitoneal lymph nodes, the peritoneum, or a distant organ [3]. Full recovery of cancer is difficult at these stages, which leads to higher mortality rates. The second reason is that ovarian cancer becomes refractory to chemotherapy after recurrence, which lowers the efficacy of therapy. One of the standard therapies for ovarian cancer is treatment with platinum drugs after surgical excision of the cancerous lesions. However, recurrence occurs in a considerable number of ovarian cancer patients, especially in the late stage and about 20–30% of these cancers show resistance to first-line chemotherapies [4].

Because of the resistance of ovarian cancer to existing chemotherapeutic agents, phytochemicals have recently received attention as potential new therapeutic agents. Coumarin and its derivatives are phytochemicals found extensively in plants. They contain benzene and pyrone ring structures and are known to have several biological activities, including antitumor effects [5,6]. Among the various coumarin derivatives, 7-hydroxycoumarin (umbelliferone) is mostly derived from Rutaceae (citrus) and Apiaceae (Umbelliferae) families. Methylation of this molecule at position four produces 4-methylumbelliferone (4-MU) [7,8]. The main function of 4-MU is hyaluronan inhibition, due to a decrease in hyaluronan synthase (HAS) 2 and 3 expression levels and, subsequently, HAS substrate (UDP-glucuronic acid) levels [9]. It has also been shown to have antitumor activity. For instance, 4-MU suppresses the growth and migration of breast cancer cells (MDA-MB-231) and markedly increases their apoptosis [10].

There is little research reported on the effects of 4-MU on epithelial ovarian cancer cells. The first research about effects of 4-MU on ovarian cancers revealed that 4-MU suppressed synthesis of hyaluronic acid in SKOV-3 human epithelial ovarian cancer cell lines via downregulation of UDP-glucuronic acids and HAS3 expressions [9]. In addition, in 2014, Tamura et al. reported that 4-MU diminished proliferation and mRNA levels of thymidine phosphorylase (TP) in HRA cell lines involved in serous subtypes and they suggested 4-MU could affect the PI3K/AKT axis because the TP levels are controlled by PI3K/AKT signaling [11,12]. However, whether 4-MU affects signaling pathway and intracellular homeostasis of epithelial ovarian cancer cell is not totally elucidated.

Therefore, this study aimed to evaluate the cytotoxic and cytostatic effects of 4-MU on the epithelial ovarian cancer cell lines ES2 and OV90 by assessing the following: (1) cell proliferation and cell cycle distribution, (2) intracellular calcium concentration, (3) levels of proteins related to endoplasmic reticulum (ER) stress, and (4) signal transduction.

## 2. Materials and Methods

### 2.1. Chemicals

4-Methylumbelliferone was purchased from Sigma-Aldrich (catalog number: M1381; St. Louis, MO, USA). Antibodies against phosphorylated AKT (Ser^473^, catalog number: 4060), S6 (Ser^235/236^, catalog number: 2211), ERK1/2 (Thr^202^/Tyr^204^, catalog number: 9101), P38 (Thr^180^/Tyr^182^, catalog number: 4511), and JNK (Thr^183^/Tyr^185^, catalog number: 4668) and for total AKT (catalog number: 9272), S6 (catalog number: 2217), ERK1/2 (catalog number: 4695), P38 (catalog number: 9212), and JNK (catalog number: 9252) were purchased from Cell Signaling Technology, Inc. (Beverly, MA, USA). Antibodies against ATF6α (catalog number: sc-166659), GRP78 (catalog number: sc-13968), GADD153 (catalog number: sc-7351), and α-tubulin (TUBA, catalog number: sc-32293) were obtained from Santa Cruz Biotechnology, Inc. (Dallas, TX, USA). The PI3K/AKT signaling pathway inhibitor LY294002 (catalog number: 9901) was purchased from Cell Signaling Technology, Inc. The ERK1/2 inhibitor U-0126 (catalog number: BML-EI282), the P38 inhibitor SB203580 (catalog number: BML-EI286), and the JNK inhibitor SP600125 (catalog number: BML-EI305) were purchased from Enzo Life Sciences, Inc. (Farmingdale, NY, USA).

### 2.2. Cell Culture

The ES2 and OV90 cell lines were obtained from the American Type Culture Collection (ATCC; Manassas, VA, USA) and were cultured in modified McCoy’s 5A medium (catalog number: 16600-082; Gibco, Carlsbad, CA, USA) containing 10% fetal bovine serum (FBS). The cells were maintained in an incubator at 37 °C under an atmosphere of 5% CO_2_. The ES2 and OV90 cells were cultured as a monolayer in 100 mm culture dishes until reaching 70–80% confluence for subsequent experiments.

### 2.3. Proliferation Assays

The ES2 and OV90 cells (3 × 10^4^ cells per 100 µL) were seeded in a 96-well plate and incubated in culture media containing 2% FBS for 12 h. After 48 h of 4-MU treatment alone (0, 0.25, 0.5, 1, 2, and 4 mM) or with inhibitors (1 mM 4-MU), cell proliferation was analyzed using a cell proliferation ELISA, BrdU kit (catalog number: 11647229001; Roche, Basel, Switzerland), in accordance with the manufacturer’s instructions. Cells were labeled with 10 µM 5-bromodeoxyuridine (BrdU) for 2 h in an incubator. They were, then, fixed for 30 min and anti-BrdU-peroxidase (POD) was added to each well. The binding of BrdU and anti-BrdU-POD was detected by assessing the reaction of POD with its substrate, 3,3′,5,5′-tetramethylbenzidine (TMB). An ELISA plate reader was used to measure the absorbance values of the reaction products at 370 nm and 492 nm.

### 2.4. Immunofluorescence Microscopy

The ES2 and OV90 cells (6 × 10^3^ cells per 300 µL) were seeded in confocal dishes and treated with 4-MU (1 mM) or left untreated for 24 h. To visualize the expression and localization of proliferating cell nuclear antigen (PCNA), cells were incubated with a mouse monoclonal anti-PCNA antibody (1:100; catalog number: sc-56; Santa Cruz Biotechnology) for 24 h after cell fixation. The cells were then incubated with goat anti-mouse IgG Alexa 488 (1:200; catalog number: A-11001; Invitrogen, Carlsbad, CA, USA) for 1 h at room temperature. Then, cells were washed three times with 0.1% bovine serum albumin (BSA) in phosphate-buffered saline (PBS). Next, we counterstained cell nuclei with 4′,6-diamidino-2-phenylindole. Images were captured using an LSM710 microscope (Carl Zeiss, Oberkochen, Germany). Fluorescence intensity was analyzed using the ImageJ program (National Health Institutes, Bethesda, MD, USA).

### 2.5. Cell Cycle Analysis

To analyze cell cycle progression, ES2 and OV90 cells (6 × 10^5^ cells per 1.5 mL) were seeded in a 6-well plate and incubated in culture medium containing 2% FBS for 12 h. then, they were treated with 0, 0.25, 0.5, or 1 mM 4-MU for 48 h. Adherent cells were detached by trypsinization for 5 min, transferred to 5 mL microcentrifuge tubes, and centrifuged to remove the culture media. Then, the cells were incubated overnight with 70% ethanol at 4 °C. They were collected by centrifugation and washed twice with 0.1% BSA-PBS. Next, the cells (100 µL) were transferred to 1.5 mL amber microtubes and resuspended in 5 µL of RNase A, before the addition of 5 µL of propidium iodide. After incubation for 30 min at room temperature, 350 µL of 1× annexin V binding buffer was added. A flow cytometer (BD Accuri C6 Plus; BD Biosciences, Franklin Lakes, NJ, USA) was used to assess the cell cycle distribution; data from three independent experiments were analyzed.

### 2.6. Measurement of Cytoplasmic Ca^2+^ Concentration

The ES2 and OV90 cells (6 × 10^5^ cells per 1.5 mL) were seeded and incubated, as described in Section 2.5. Adherent cells were detached by trypsinization for 5 min and pelleted by centrifugation. Pellets were resuspended in 3 µM Fluo-4 AM (Invitrogen), diluted with culture medium, and incubated in an incubator at 37 °C and 5% CO_2_ conditions. Cells were washed with cold PBS after 20 min of incubation. A flow cytometer (BD Accuri C6 Plus; BD Biosciences) was used to detect Fluo-4 AM-stained cells; data from three independent experiments were analyzed.

### 2.7. Measurement of Mitochondrial Ca^2+^ Concentration

The ES2 and OV90 cells (6 × 10^5^ cells per 1.5 mL) were seeded and incubated, as described in Section 2.5. Cells were detached by incubation in trypsin-EDTA and were pelleted by centrifugation. Pellets were resuspended in 3 µM Rhod-2 AM (Invitrogen), diluted in cold Hank’s balanced Salt Solution (HBSS) without Ca^2+^ or Mg^2+^ (Gibco), and incubated at 4 °C. After 30 min of incubation, cells were washed with 400 µL of prewarmed HBSS. A flow cytometer (BD Accuri C6 Plus; BD Biosciences) was used to detect Rhod-2 AM-stained cells; data from three independent experiments were analyzed.

### 2.8. Western Blot Analyses

To determine the protein expression levels, the ES2 and OV90 cells were treated with 0, 0.25, 0.5, or 1 mM 4-MU and lysed with lysis buffer containing Triton X-100 (catalog number: X100; Sigma-Aldrich). The Bradford protein assay (Bio-Rad, Hercules, CA, USA) was performed to measure the protein concentration in whole-cell lysates. After denaturation, proteins were separated by 10% sodium dodecyl sulfate-polyacrylamide gel electrophoresis and transferred to a nitrocellulose membrane. Each membrane was, then, incubated with primary antibodies (1:1000). An enhanced chemiluminescence detection solution (Super Signal West Pico; Pierce, Rockford, IL, USA) was used to develop the blots and a ChemiDoc EQ system and Quantity One software (Bio-Rad) were used to detect and quantify the chemiluminescence intensity of the protein bands. The intensity of the target proteins in the blots was normalized using the intensity of total proteins or that of α-tubulin (TUBA). Data from three independent experiments were analyzed according to the statistical analysis methods described below.

### 2.9. Statistical Analysis

All data were analyzed by analysis of variance in accordance with the general linear model of the Statistical Analysis System software (SAS Institute, Inc., Cary, NC, USA), to confirm whether the effects of 4-MU on epithelial ovarian cancer cells were statistically significant. A *p*-value ≤ 0.05 was considered to be statistically significant. Data are shown as the means ± standard deviations.

## 3. Results

### 3.1. 4-MU Suppressed Ovarian Carcinoma Cell Proliferation through G2/M Phase Arrest Cells

**Figure 1:**
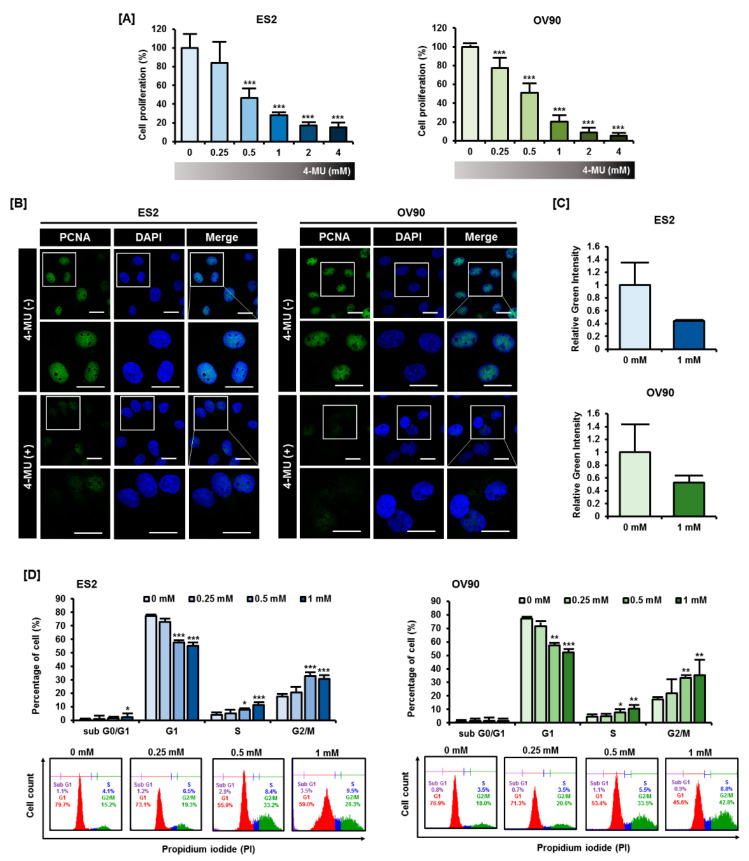
Effects of 4-methylumbelliferone (4-MU) on ES2 and OV90 cell proliferation. (**A**) A BrdU cell proliferation assay was performed to measure the anti-proliferative effects of 4-MU (0, 0.25, 0.5, 1, 2, 4 mM) on ES2 and OV90 cells. Cell proliferation in the 4-MU-treated group was calculated as a percentage relative to that in the vehicle-treated group; (**B**) PCNA localization (green) in the nucleus was detected by confocal laser microscopy and 4′,6-diamidino-2-phenylindole (DAPI, blue) counterstaining was used to visualize the nuclei. Scale bar, 20 µm; (**C**) Green fluorescence intensity was quantified using ImageJ and comparative green intensity of 4-MU treated groups was represented as compare with vehicle-treated groups; (**D**) The effect of 4-MU on cell cycle progression was determined using propidium iodide (PI) staining and flow cytometry in ES2 and OV90 cells. The percentage of cells in each phase was calculated based on the total cell population.

We assessed the effects of 4-MU on epithelial ovarian cancer cell proliferation. To identify the effects of 4-MU on various subtypes of epithelial ovarian cancer, we selected the ES2 cell line, clear cell carcinoma, and OV90 cell line, high-grade serous carcinoma, in this study. Firstly, we performed proliferation assays by measuring BrdU incorporation in ES2 and OV90 cells and found that 4-MU significantly decreased the growth of ES2 and OV90 cells in a dose-dependent manner (Figure 1A). In particular, 1 mM 4-MU decreased the proliferation of ES2 cells and OV90 cells to approximately 28% (*p* < 0.001) and 20% (*p* < 0.001), respectively, of that of the vehicle-treated cells. Because 4-MU effectively decreased ovarian cancer cell proliferation at a concentration of 1 mM, we further investigated the expression and localization of PCNA, which is involved in DNA replication, in ES2 and OV90 cells treated with 1 mM 4-MU. In both cell lines, the intensity of PCNA staining decreased to approximately half of the intensity observed in vehicle-treated cells following 4-MU treatment (Figure 1B,C). Because PCNA is highly associated with cell cycle progression, we next evaluated cell cycle progression using flow cytometry (Figure 1D). The ES2 and OV90 cells were found to be arrested at the G2/M phase following 4-MU treatment. The ratio of cells accumulated in the G1 phase decreased, whereas the number of G2/M phase cells increased by an average of approximately 1.7-fold for ES2 cells (*p* < 0.001) and 2-fold for OV90 (*p* < 0.01) cells as compared with the vehicle-treated cells. Collectively, these results indicated that 4-MU inhibited the proliferation of ES2 and OV90 cells by inducing G2/M arrest.

### 3.2. 4-MU Induced a Perturbation of Intracellular Calcium Homeostasis

**Figure 2:**
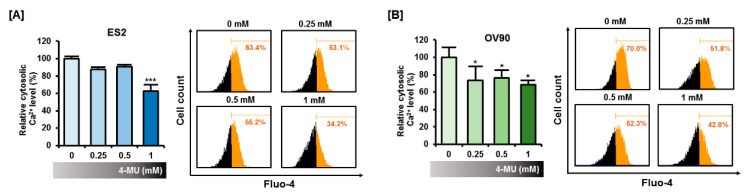
Effects of 4-MU on cytoplasmic calcium concentration in ES2 (**A**) and OV90 (**B**) cells. Cytoplasmic calcium concentration was measured by flow cytometry using Fluo-4 AM and data were quantified relative to the calcium level of the vehicle-treated group. Each experiment was performed in biological triplicates. Flow cytometry histograms from one of the three experiments are presented. * *p* < 0.05 and *** *p* < 0.001, for vehicle-treated vs. 4-MU-treated groups.

Because intracellular calcium ion serves as a regulator of several cellular processes including the progression of cell cycle, [13] we investigated whether 4-MU disrupts intracellular calcium homeostasis. Thus, we measured calcium levels in vehicle-treated and 4-MU-treated cells via flow cytometry. Cytoplasmic calcium concentration ([Ca^2+^]_c_) was determined by staining with the Fluo-4 AM dye (Figure 2A,B). In the ES2 cells, a significant reduction in [Ca^2+^]_c_ occurred after treatment with 1 mM 4-MU (*p* < 0.001), whereas in OV90 cells, [Ca^2+^]_c_ was reduced by 4-MU concentrations starting from 0.25 mM (*p* < 0.05). In the 4-MU-treated cells, calcium levels decreased to approximately 60% of the calcium levels of vehicle-treated cells. This result revealed that 4-MU interfered with intracellular calcium homeostasis. In addition, we speculated that 4-MU could influence organelles related to calcium homeostasis such as the ER and mitochondria.

### 3.3. 4-MU Disrupted the Homeostasis of Cellular Organelles in Epithelial Ovarian Cancer Cells

**Figure 3:**
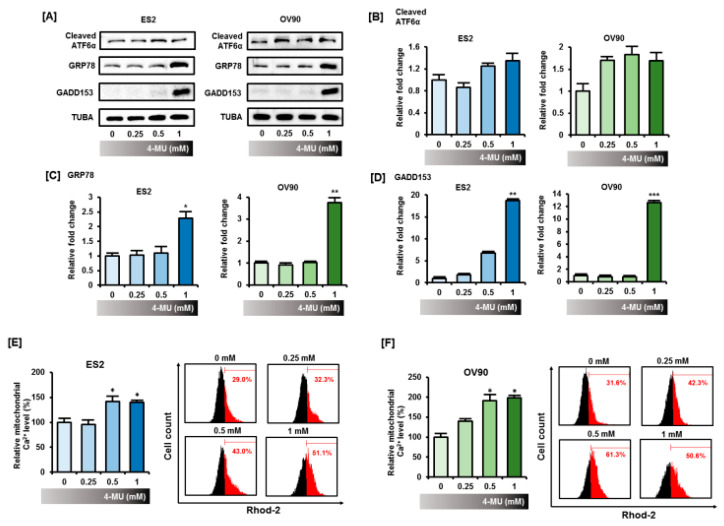
4-MU induced ER stress and increased mitochondrial calcium levels in ES2 and OV90 cells. (**A**) Protein expression levels of cleaved ATF6α, GRP78, and GADD153 were determined by Western blot analyses; The relative intensity of the Western blot bands of cleaved ATF6α (**B**), GRP78 (**C**), and GADD153 (**D**) were normalized to the total protein levels. The relative intensity was averaged over three independent experiments; (**E**,**F**) Calcium concentration in mitochondria was measured by flow cytometry using Rhod-2 AM. Each experiment was performed in triplicate and flow cytometry histograms from one of the three experiments are presented. * *p* < 0.05, for untreated vs. 4-MU treated groups.

Next, we investigated the effects of 4-MU on ER stress by analyzing the expression levels of the ER stress-related proteins cleaved activating transcription factor 6α (ATF6α), 78-kDa glucose-regulated protein (GRP78), and growth arrest- and DNA damage-inducible protein 153 (GADD153). As shown in Figure 3A, ER stress protein expression levels in the ES2 and OV90 cells were significantly increased by 4-MU treatment. The increase in cleaved ATF6α levels was not dose-dependent, but they were slightly elevated after 4-MU treatment (Figure 3B). The expression levels of GRP78 and GADD153 after treatment with 1 mM 4-MU showed a great increase as compared with those in untreated cells (Figure 3C,D). Since the ER is closely associated with the maintenance of mitochondrial calcium homeostasis, we stained ES2 and OV90 cells with the mitochondrial calcium indicator Rhod-2 AM. As shown in Figure 3E,F, the mitochondrial calcium concentration ([Ca^2+^]_mt_) significantly increased (*p* < 0.05) in ES2 cells after treatment with 4-MU. After treating OV90 cells with 1 mM 4-MU, [Ca^2+^]_mt_ almost doubled as compared with [Ca^2+^]_mt_ in the vehicle-treated cells (*p* < 0.05). Taken together, these results indicated that 4-MU treatment disrupted organelle homeostasis, specifically increasing ER stress and mitochondrial calcium levels.

### 3.4. 4-MU Downregulated PI3K/AKT Signaling and Upregulated MAPK Signaling

**Figure 4:**
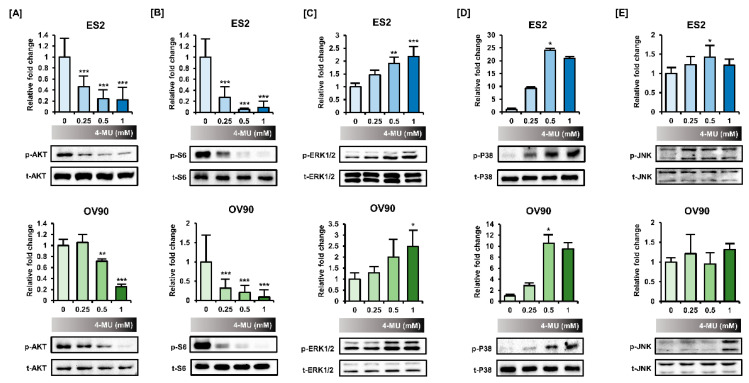
Regulation of PI3K/AKT and MAPK signaling by 4-MU. Phosphorylation levels of AKT (**A**), S6 (**B**), ERK1/2 (**C**), P38 (**D**), and JNK (**E**) were determined by Western blot analyses after 4-MU treatment. The relative intensity was averaged over three independent experiments and was normalized to the total protein levels. * *p* < 0.05, ** *p* < 0.01, and *** *p* < 0.001, for untreated vs. 4-MU treated groups.

Next, the phosphorylation levels of PI3K/AKT and MAPK signaling proteins were analyzed via Western blot analyses to determine whether 4-MU influenced signaling transduction in ES2 and OV90 cells. The relative fold change of the phosphorylated protein level is shown in Figure 4 and the ratio of phosphorylated/non-phosphorylated proteins is shown in Table 1. The AKT phosphorylation levels decreased following 4-MU treatment at concentrations from 0.25 mM in ES2 cells and from 0.5 mM in OV90 cells (Figure 4A). In addition, the S6 phosphorylation levels decreased markedly in both cell types (Figure 4B). However, the phosphorylation levels of all MAPK proteins increased in the 4-MU treated cells. The ERK1/2 phosphorylation levels gradually increased in ES2 and OV90 cells after 4-MU treatment (Figure 4C). The phosphorylated P38 levels were relatively low in untreated cells but were enhanced in cells treated with 0.5 mM and 1 mM 4-MU (Figure 4D). Slight increases in JNK phosphorylation levels were also observed after 4-MU treatment (Figure 4E).

**Figure 5:**
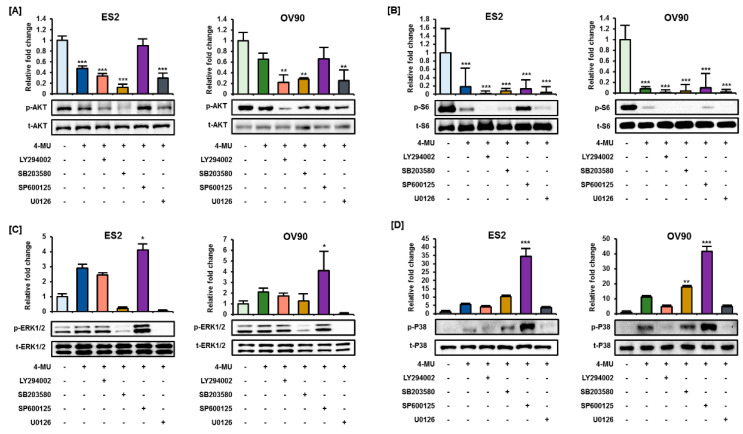
Pretreatment with pharmacological inhibitors before 4-MU treatment alters the phosphorylation of signaling proteins. Phosphorylation levels of AKT (**A**), S6 (**B**), ERK1/2 (**C**), and P38 (**D**) in ES2 and OV90 cells were measured by Western blot analyses. The relative intensity of the bands of each protein was normalized to the total protein levels and was averaged over three independent experiments. * *p* < 0.05, ** *p* < 0.01, and *** *p* < 0.001, for untreated vs. 4-MU treated groups.

To determine how 4-MU regulates these intracellular signaling pathways, we pretreated cells with 20 μM of the pharmacological inhibitors LY294002 (PI3K inhibitor), SB203580 (P38 inhibitor), SP600125 (JNK inhibitor), or U0126 (ERK1/2 inhibitor) prior to 4-MU (1 mM) treatment, and then performed Western blot analyses. Phosphorylated AKT and S6 levels showed a greater decrease after combined treatment of ES2 cells with 4-MU and LY294002, SB203580, or U0126 as compared with 4-MU treatment alone. However, the decreased phosphorylation levels in 4-MU-treated ES2 cells were recovered by SP600125 pretreatment (Figure 5A,B). Unexpectedly, ERK1/2 phosphorylation levels were significantly decreased by U0126, and also SB203580 (Figure 5C). Moreover, U0126 and LY294002 decreased the P38 phosphorylation levels in ES2 and OV90 cells (Figure 5D).

### 3.5. The Combination of 4-MU and Pharmacological Inhibitors Had Synergistic Anti-Proliferative Effects

**Figure 6:**
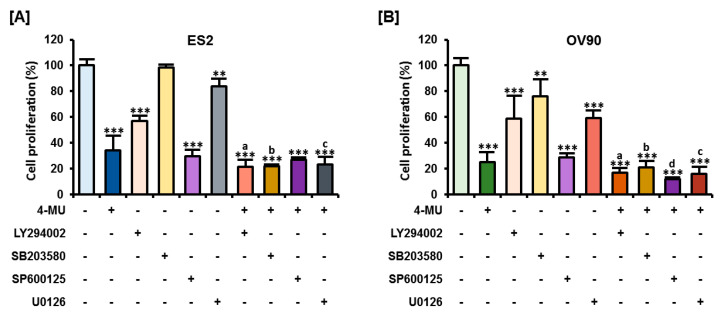
Effects of the co-treatment with 4-MU and pharmacological inhibitors on human ovarian cancer cells. Cells were incubated with 20 μM LY294002, SB203580, or SP600125 or 10 μM U0126, with or without 4-MU (1 mM). Cell proliferation assays were performed as previously described for ES2 (**A**) and OV90 (**B**) cells. ** *p* < 0.01 and *** *p* < 0.001, for vehicle-treated vs. and 4-MU treated groups. Each letter indicates significant differences between the inhibitor with or without 4-MU. a, significant difference between LY294002 treatment alone and combined 4-MU/LY294002 treatment; b, significant difference between SB203580 treatment alone and combined 4-MU/SB203580 treatment; c, significant difference between U0126 treatment alone and combined 4-MU/U0126 treatment; d, significant difference between SP600125 treatment alone and combined 4-MU/SP600125 treatment.

The pharmacological inhibitors used to test signaling pathways in the previous experiments also have antitumor effects. Thus, we assessed the synergistic effects of 4-MU and these pharmacological inhibitors on ovarian cancer cell proliferation. In ES2 cells, all inhibitors, except SB203580, decreased the number of proliferating cells when used alone (Figure 6A). Combined treatments with 4-MU and LY294002, SP600125, or U0126, significantly reduced the number of proliferating ES2 cells as compared with treatments using the inhibitors alone. All inhibitors, including SB203580, decreased the percentage of proliferating OV90 cells (Figure 6B). All combinations of 4-MU and these inhibitors showed a greater reduction in the number of proliferating OV90 cells than treatment with each of the inhibitors alone. However, only LY294002, SB203580, and U0126 had a synergistic effect with 4-MU in decreasing ES2 cell proliferation.

## 4. Discussion

**Figure 7:**
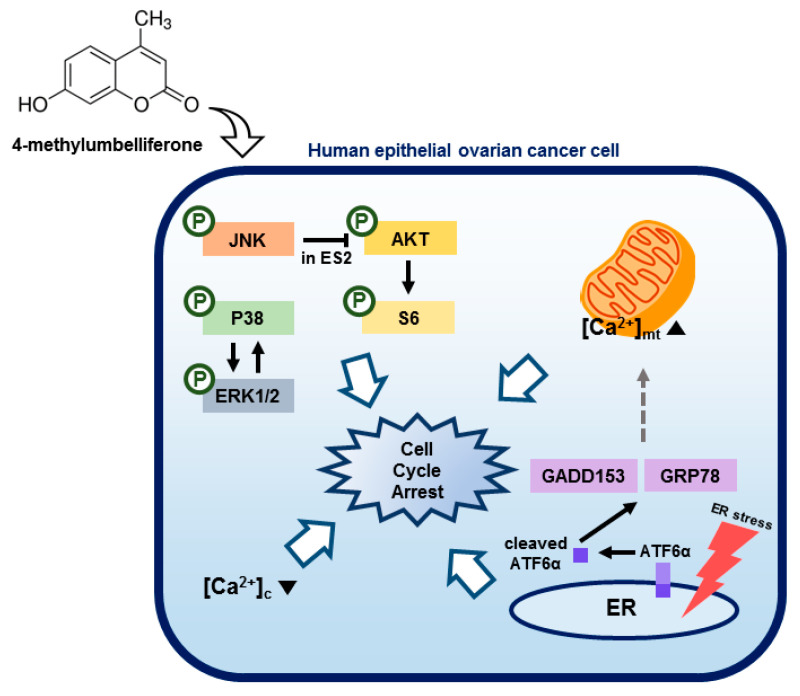
Illustration of the hypothetical action of 4-MU on intracellular calcium levels, the endoplasmic reticulum (ER), and signal transduction in human epithelial ovarian cancer cells. [Ca^2+^]_c_, cytoplasmic calcium concentration; [Ca^2+^]_mt_, mitochondrial calcium concentration.

The intracellular mechanisms responsible for the downregulation of epithelial ovarian cancer cell proliferation by 4-MU are unknown. We demonstrated that 4-MU reduced the proliferation of epithelial ovarian cancer cells (ES2 and OV90) by inducing G2/M phase arrest. Moreover, 4-MU affected intracellular calcium levels, ER homeostasis, and PI3K/AKT and MAPK signaling pathways. The combination of 4-MU and pharmacological inhibitors effectively suppressed cancer cell proliferation. The effects of 4-MU on ES2 and OV90 cells are summarized in Figure 7.

The antitumor effects of 4-MU, a well-known hyaluronan inhibitor, on various cancer types have previously been reported. For example, 4-MU suppressed the proliferation of human breast cancer, pancreatic cancer, and prostate cancer cells [10,14,15]. Moreover, 4-MU caused the arrest of cancer cells at the G2/M phase of the cell cycle in canine mammary tumors [16], which is similar to our findings in ovarian cancer cells. Since modulating the cell cycle progression of cancer cells has been reported as an effective therapeutic method, several drugs have been used to induce cell cycle arrest in ovarian cancer. Paclitaxel, which is widely used for cancer treatment, disrupts the microtubule dynamics that are needed for the normal segregation of chromosomes. This leads to G2/M phase arrest via the mitotic checkpoint system and it induces the apoptosis of cancer cells [17,18]. Moreover, previous studies have indicated that many natural extracts, such as *Duchesnea indica* (Andr.) Focke, genistein, and daidzein could suppress ovarian cancer cell growth by inhibiting cell cycle progression [19,20,21]. Therefore, the cytostatic function of 4-MU can also be utilized for ovarian epithelial cancer treatment.

Intracellular calcium is an essential molecule for determining cell fate; it mediates several signaling pathways, including inositol 1,4,5-trisphosphate, MAPK, cyclic AMP (cAMP), and calmodulin pathways [22]. In the present study, the depletion of cytoplasmic Ca^2+^ was observed after 4-MU treatment. Although there are many reports that have suggested that increased [Ca^2+^]_c_ induced cell death, a reduction in [Ca^2+^]_c_ could also exert cytotoxic effects. For instance, lowering [Ca^2+^]_c_ using calcium channel antagonists has been reported to suppress HT-39 (breast cancer cell line) cell growth [23]. In epithelial ovarian cancer cells, a reduction in [Ca^2+^]_c_ induced by gentisyl alcohol resulted in decreased cell viability and increased apoptosis [24]. Moreover, some factors downstream of calcium signaling can affect the cell cycle. For example, CaMKII, which is activated after Ca^2+^/calmodulin binding, regulates cell cycle progression [25]. Phosphorylation of cell division cycle 25c (cdc25c) by CaMKII facilitates cell cycle progression, whereas G2/M phase arrest occurs in HeLa cells following treatment with a CaMKII inhibitor [26]. Similarly, CaMKII inhibitory protein alters the expression of proteins associated with cell cycle regulation and arrests cells at the G0/G1 phase in ovarian adenocarcinoma cell lines [27]. Even if the precise mechanisms by which 4-MU affects the cell cycle are not fully discovered, these prior studies have suggested that the reduction in intracellular calcium levels by 4-MU could be one of the causes of the G2/M arrest of epithelial ovarian cancer cells.

Increased concentrations of unfolded proteins in the ER can provoke ER stress and the unfolded protein response (UPR) [28]. Here, we observed elevated levels of cleaved ATF6α, GRP78, and GADD153 after 4-MU treatment. ATF6α, which is one of the UPR signaling activators, converts to the cleaved form following ER stress. Fragmented ATF6α can regulate the transcription of various UPR target genes, including *GRP78* and *GADD153* [29]. This response can trigger cell death. For example, increased expression levels of GRP78 and GADD153 decrease the viability and promote the apoptosis of ovarian cancer cells (SKOV3) [30]. [Ca^2+^]_mt_ can be elevated by alterations in calcium flux between the ER and mitochondria, which occur as a result of UPR and can contribute to cell death via apoptosis and autophagy [31,32]. A breakdown in homeostasis in the ER and mitochondria, induced by 4-MU treatment, could serve as an antitumor mechanism.

The regulation of signal transduction is a key therapeutic method in cancer treatment. We found that 4-MU decreased the phosphorylation levels of AKT and S6. The PI3K/AKT pathway is a well-known pathway that is closely associated with cell cycle regulation. In the epithelial ovarian cancer A2780 cells, p21^Cip1^ is a downstream factor of the PI3K/AKT pathway and inactivation of AKT reduces p21^Cip1^ expression levels [33]. However, p21^Cip1^ interacts differently with cyclin, cyclin-dependent kinases, and PCNA according to each cell cycle phase during normal cell cycle progression [34]. Hence, we assumed that the hypo-phosphorylation of AKT induced by 4-MU could be responsible for cell cycle arrest. Contrary to this assumption, the phosphorylation levels of two MAPKs, ERK1/2 and P38, were increased following 4-MU treatment. P38 is known to be activated in stress conditions and it usually has antitumorigenic effects, which is consistent with the results of our present study [35]. Although ERK1/2 shows context-dependent effects on cell survival, there are a number of studies that have shown that activated ERK1/2 decreased cancer cell viability. For instance, a Chinese bayberry-derived flavonoid decreased ovarian carcinoma cell viability through an ERK-dependent apoptotic pathway [36]. Furthermore, increased ERK1/2 phosphorylation levels can induce autophagy, senescence, and reactive oxygen species production in various cell types [37]. For a more comprehensive understanding of the signaling pathway involved in mediating the effects of 4-MU, we incubated ES2 and OV90 cells with a combination of 4-MU and pharmacological inhibitors. We found that 4-MU slightly increased JNK phosphorylation levels and inhibited AKT and S6 phosphorylation. In ES2 cells, treatment with the JNK inhibitor SP600125 promoted AKT and S6 phosphorylation. Taking these two results together, we proposed that the increased levels of phosphorylated JNK, induced by 4-MU, suppressed the phosphorylation of AKT and S6 in ES2 cells. Furthermore, 4-MU increased the levels of phosphorylated ERK1/2 and P38. The ERK1/2 inhibitor U0126 suppressed the phosphorylation of P38, and the P38 inhibitor SB203580 suppressed ERK1/2 phosphorylation. Therefore, we hypothesized that phosphorylated ERK1/2 and P38 activate each other in 4-MU-treated cells. Although the adequate dose and duration of the clinical application of 4-MU for cancer treatment is not yet established, there are many previous studies, in animals and humans, that have indicated that 4-MU could have clinical uses. Currently, 4-MU is being used to treat biliary dyskinesia in Europe and Asia and no side effects have been reported after the oral administration of 4-MU (1200 mg/day) for three months in biliary dyskinesia patients [7,38].

## 5. Conclusions

In conclusion, we demonstrated the anti-proliferative effects of 4-MU on human epithelial ovarian cancer cells (ES2 and OV90) through G2/M arrest. Moreover, disruption of intracellular homeostasis, including a decrease in [Ca^2+^]_c_, increased ER stress, and an increase in [Ca^2+^]_mt_, as well as an alteration in signal transduction pathways, were triggered by 4-MU. Because of the efficacy of 4-MU treatment on ovarian carcinoma cell lines and its previously demonstrated safety in clinical trials, 4-MU could be a promising therapeutic agent for use in epithelial ovarian cancer patients.

## Figures and Tables

**Table 1:** The ratio of phosphorylated/non-phosphorylated proteins in ES2 and OV90. Data are presented as average ± SD over three independent experiments.

**ES2**	**p-AKT**	**p-S6**	**p-ERK1/2**	**p-P38**	**p-JNK**
0 mM	1.58 ± 0.33	0.68 ± 0.32	0.47 ± 0.12	0.02 ± 0.01	0.97 ± 0.14
0.25 mM	0.73 ± 0.18	0.20 ± 0.18	0.68 ± 0.17	0.10 ± 0.03	1.19 ± 0.22
0.5 mM	0.39 ± 0.17	0.03 ± 0.01	0.90 ± 0.25	0.46 ± 0.52	1.36 ± 0.29
1 mM	0.32 ± 0.23	0.08 ± 0.10	1.03 ± 0.36	0.39 ± 0.36	1.17 ± 0.14
**OV90**	**p-AKT**	**p-S6**	**p-ERK1/2**	**p-P38**	**p-JNK**
0 mM	0.32 ± 0.10	1.62 ± 0.69	0.62 ± 0.25	0.10 ± 0.06	0.95 ± 0.13
0.25 mM	0.35 ± 0.13	0.50 ± 0.22	0.78 ± 0.31	0.32 ± 0.35	1.21 ± 0.52
0.5 mM	0.22 ± 0.03	0.31 ± 0.18	1.31 ± 0.78	1.08 ± 1.40	0.95 ± 0.30
1 mM	0.08 ± 0.04	0.16 ± 0.17	1.43 ± 0.72	1.10 ± 1.05	1.32 ± 0.18
